# With open science gaining traction, do we need an Australasia PubMed Central (PMC)? A qualitative investigation

**DOI:** 10.1371/journal.pone.0212843

**Published:** 2019-02-22

**Authors:** Lisa M. Kruesi, Frada V. Burstein, Kerry J. Tanner

**Affiliations:** Faculty of Information Technology, Monash University, Melbourne, Victoria, Australia; Lancaster University, UNITED KINGDOM

## Abstract

Open biomedical repositories, such as PubMed Central (PMC), are a means to make research discoverable and permanently accessible. Assessing the potential interest of key stakeholders in an Australasia PubMed Central was the objective of this research. The investigation is novel, assisting in the development of open science infrastructure through its systematic analysis of the potential interest in, and viability of a biomedical repository for managing openly accessible research outputs for the Australasia region. The research adopted a qualitative approach based on semi-structured interviews and a focus group. Forty-four stakeholders located throughout Australia and New Zealand participated in the research. Participants expanded upon their experience of PubMed, MEDLINE, PMC and their use of information resources for research and clinical practice. The Evidence Based Healthcare (EBHC) pyramid was the theoretical model adopted to explain open biomedical repository processes. A strengths, weaknesses, opportunities and threats (SWOT) analysis identified support for exploring membership of an international PMC system, in particular Europe PMC. Lessons learnt from PMC US, Europe PMC and PMC Canada (collectively known as PubMed Central International) informed the investigation. A major strength identified was that PubMed Central International has been able to achieve high levels of compliance way beyond that of most institutional repositories. A great threat faced is overcoming the difficulties of working together with other major world bodies and financially sustaining an Australasia PMC. Improving Australasian biomedical knowledge management processes may be possible from adopting a PMC for retrieving and transferring research, linked to the data underlying the research. This in turn could help put regional research under a brighter spotlight, potentially leading to improvements in research quality. There is an opportunity for a potential Australasia PMC to harvest biomedical research from the National Library of Australia’s aggregator database, Trove and work closely with Europe PMC to avoid duplication of effort. Overall, establishment of an Australasia permanent biomedical digital open repository is perceived as important, with significant potential flow-on benefits to healthcare, industry and society.

## Introduction

Open biomedical research on an international scale is gaining traction. Working with the parent site, PubMed and PubMed Central (PMC), established by the US National Library of Medicine, mirror PMC repositories in Europe and Canada were formed as disciplinary repositories, to make world biomedical research and related data permanently accessible and discoverable[1]. In 2018, PMC Canada was taken offline, whereas the Europe PMC site continues to develop features and functionality [2,3].

The US National Library of Medicine, creator of PubMed, established an open biomedical and life sciences repository of freely accessible full-text journal literature PMC in 2000. PubMed is an aggregator database, the precursor of which was the printed Index Medicus that began in 1879 [4]. PMC, as an open biomedical repository, enables the publications resulting from the National Institutes of Health’s funded research to be openly and permanently available from the NLM’s website. According to Richard Roberts, executive editor of *Nucleic Acids Research* and Nobel Prize winner for medicine in 1993, the goal of PMC is not to replace the journal; the objective of a PMC is to distribute knowledge as widely as possible [5]. At the time of this research, the US NLM PMC was the parent site to two PMC sites established as PMC International members. Europe PMC and PMC Canada’s role was to expand the participation in the PMC repository and add to the increasing corpus of open access research output [6,7].

Motivation for this research comes from a community of practice of librarians that has been active over the past three decades. A proposed Australasia PMC has the potential to achieve a number of goals. Reducing the duplication of effort, and the fragmented and incomplete access to health research output that presently exists with institutional repositories are major incentives. Establishing a PMC for sites without repositories and expanding the corpus of knowledge within PMC International are other goals. In addition, preserving health research and associated data for present and future generations of users throughout the world by becoming a node of an internationally proven PMC system, which produces quality metadata that is widely discoverable, are other important incentives.

This paper reports on a strengths, weaknesses, opportunities and threats (SWOT) analysis of a potential Australasia PMC. Assessing the potential interest of key stakeholders in an Australasia PubMed Central was the objective of this research. The investigation contributes to the development of open science infrastructure through its systematic analysis of the potential interest in, and viability of a biomedical repository for managing openly accessible research outputs for the Australasia region. The ‘Australasia region’ for this research includes Australia and New Zealand. Developments in scholarly communications and digital infrastructure, along with a lack of previous reports on the topic make this work useful and timely.

Interviews and a focus group conducted with key Australian and New Zealand stakeholders revealed the strengths and weaknesses, opportunities and threats related to joining PMC International (PMCI) and establishing an Australasia consolidated medical and health sciences research repository.

Institutional repositories throughout Australasian universities exist to manage and disseminate research output. For example, from 2007–2009, the Australian Government administered the Australian Scheme for Higher Education Repositories (ASHER) program, during which Australian universities received AUD$26 million for ongoing development of institutional repositories. ‘Enhancing access to research through the use of digital repositories’ was the aim of ASHER, though at the time much of the allocation was assigned to developing closed collections for the Excellence in Research Australia (ERA) project [8]. Clifford Lynch, Executive Director of the Coalition for Networked Information, argues that ‘to date, the most successful repositories have tended to be disciplinary or national/regional in scope rather than institutional repositories [9]. Even so, institutional and disciplinary repositories continue to co-exist [10].

## Methods

### Research design

Stakeholder interest in an investigation on the concept, viability and potential for an Australasia open biomedical repository is the proposition expanded upon, as the first cycle of an action research project. Action research is particularly relevant for practitioner research because it involves the people who are experiencing the organizational or social challenges being addressed [11]. The dual aims of action research are for practical problem solving, and for testing and/or potentially developing a model or theory. The EBHC pyramid and KM models were used as the theoretical lenses for this research. The change intervention, which is a key component of action research, comprises the recommendations made by the Australasia PMC Working Group [12]. The data collection, research techniques included semi-structured interviews and a focus group.

#### Research team

The first author of this paper was the interviewer with thirty years of experience as a biomedical librarian working in a major national research organisation and three leading Australian research universities. The other authors of this paper provided expertise in designing the study and assisting with the conceptual analysis of the data from the interviews. The research commenced in 2016. Members of an Australasia PMC Working Group (originally set up as an Australia PMC Steering Committee to provide advice and feedback on an Australia PMC investigation) also gave feedback on the draft interview questions. When a member joined the PMC Working Group from the University of Auckland, the name of the group became the Australasia PMC Working Group. A senior librarian pilot tested the interview questions. The Monash University Human Research Ethics Committee approved the study and the associated documentation.

### Study design

The Evidence Based Healthcare (EBHC) pyramid, developed by Haynes in 2001, was the theoretical model adopted to explain the use of research output contained in a PMC [13]. The EBHC has been widely adopted to help determine which resources to consult for answering clinical queries and to provide a perspective on the wide array of health sciences information resources available from library databases and repositories [14].

The research used purposive sampling to seek comprehensive and authoritative feedback on the proposal to establish an Australasia PMC. Research participants were either colleagues or known by their professional reputation to the interviewer. As shown in Table 1, the selection of participants ensured gender diversity and geographic spread across Australia. The research team chose potential stakeholders of an Australasian PMC based on their professional roles as leaders in their field, to participate in the research. Participant representation from fields of biomedical research, clinical practice and healthcare organisations, medical societies, publishing, universities and libraries was required. Participants each received a study explanatory statement, a briefing document and interview questions prior to either an interview or the focus group. All potential 17 stakeholders accepted their interview invitation and the 28 health sciences librarians consented to participating in a focus group session. The 28 health sciences librarians included 20 working in university libraries and 8 working in hospital libraries located throughout Australia. The focus group took place on the last day of a four day, Evidence Based Practice Librarians’ residential workshop.

**Table 1:** Summary of the participants.

No	Occupation	Gender	Location of interviewees	Colleague of the interviewer	Type of session
**Group 1. Biomedical Researchers**						
1	Head biomedical researcher	Male	Queensland, Australia	No	Zoom interview
2	Head biomedical researcher	Male	Victoria, Australia	Yes	Meeting on site
**Group 2. Senior Executives and Open Access Leaders**						
3	Senior executive (leader in open access)	Female	Queensland, Australia	Yes	Zoom interview
4	Senior executive (leader in open access)	Male	Queensland, Australia	No	Zoom interview
5	Senior executive (leader in open access)	Male	Queensland, Australia	Yes	Zoom interview
6	Senior executive (leader in open access)	Female	Canberra, Australia	No	Zoom interview
7	Senior executive & biomedical researcher (leader in open access)	Female	Victoria, Australia	No	Zoom interview
**Group 3. Clinicians**						
8	Hospital clinician & editor in chief of a medical journal	Male	North Island, New Zealand	No	Zoom interview
9	Clinician & academic	Male	New South Wales, Australia	Yes	Phone interview
10	Clinician & academic	Male	Queensland, Australia	Yes	Zoom interview
11	Nursing academic	Female	New South Wales, Australia	Yes	Zoom interview
12	Hospital clinician & allied health practitioner	Female	Victoria, Australia	Yes	Zoom interview
**Group 4. Health Sciences Librarians and Repository Managers**						
13	Senior repository manager	Male	Western Australia, Australia	No	Zoom interview
14	Senior repository manager	Male	Victoria, Australia	Yes	Zoom interview
15	External relations, medical society	Female	Victoria, Australia	No	Zoom interview
16	Senior librarian	Female	New South Wales, Australia	Yes	Zoom interview
17	Senior librarian	Female	Western Australia, Australia	Yes	Zoom interview
18–45	Health sciences librarians (28)	Mixed	South Australia, Australia	No	Focus Group

The semi-structured interviews and the focus group occurred between December 2016 and February 2017. Interview questions related to participants’ experience of PubMed, MEDLINE, PMC and their use of information resources for research and clinical practice [S1 interview and focus group questions]. The 45 participants included two Head biomedical researchers, a senior executive from the Australian Research Council (ARC), a senior executive and biomedical researcher from Australia’s National Health and Medical Research Council (NHMRC), executive directors, prominent clinical academics and practitioners, university repository managers, medical library leaders located throughout Australia and a medical journal editor and retired hospital director from New Zealand. According to Creswell, a sample size of 20–30 is adequate to obtain feedback for most or all perceptions and to achieve saturation on the topic. The sample here generated a sufficiently informative range of opinion on the subject matter [15].

All participants contacted agreed to partake in the interview and there were no participant dropouts. Interviews were in person or captured using the Zoom video conferencing system. All of the interviews took place in a workplace setting and took approximately one hour each. Twenty-eight health sciences librarians provided input in a focus group setting to the same questions answered by interview participants. Consent authorisation for use of the interview findings was obtained. NVivo software was used by the authors for coding the interview transcripts in the form of a SWOT analysis.

### SWOT analysis

Themes identified from the interview transcripts were coded as Strengths or Opportunities towards the establishment of an Australasia PMC or Weaknesses and Threats against the establishment of a potential Australasia PMC. Table 2 is a summary of the major themes identified.

**Table 2:** Strengths & weaknesses, opportunities & threats analysis.

**PMCI Strengths & Weaknesses**	**Strengths**	**Weaknesses**
	1. Established PMC system	1. Many clinicians do not read primary research
	2. Research linked to grant details	2. Information overload
	3. Means to achieve compliance with funding bodies	3. Adequately served by existing resources
	4. Promote repository services & expertise	4. No guarantee of funding or means to ensure longevity of a PMC
	5. Encourage retention of intellectual property by researchers	
	6. Consolidation of international biomedical research	
	7. Remove pay-wall to quality research	
	8. Importance of primary resources to researchers	
	9. Get information out of research silos	
**Australasia PMC Opportunities & Threats**	**Opportunities**	**Threats**
	1. Regional content	1. Institutional repositories adequately meet present needs
	2. Increasing the availability & number of Australasian biomedical open access research papers, along with synthesized & filtered content	2. Need for a national body to make a long term commitment to establish & fund a PMC
	3. Lobby for regional needs & desired features in PubMed/PMCI	3. Inability for sectors to work together to establish & manage a PMC
	4. Access to repository expertise & system features	4. Convenience of present access to university online journal subscriptions to eligible clinician researchers
	5. Reduce gap between translation of research into practice	
	6. Make content more discoverable	
	7. Foundation of Australasia Medical Library	
	8. Establish online collections to complement PMC	
	9. Means to raise quality of research	
	10. Source for engagement & impact evidence	
	11. Consolidate & integrate data-sets & increase mineable content	
	12. Partner with PMCI & national libraries	

### Strengths

For biomedical researchers PubMed, MEDLINE and PMC are the foundation, primary research repositories. As funders, such as the ARC and the NHMRC, have open access policies that require researchers adhere to openly publishing articles, considering an Australasian PMC for reporting on research performance is a means to achieve funding body compliance. Institutional executives and open access leaders view the PMC system, such as the Europe PMC, as a means to manage and review the output of biomedical research linked to grant details and a means to help avoid duplication of research and link related findings.

Health sciences librarians and biomedical researchers commented that an Australasia PMC might be a way of reducing the fragmentation of university repository systems by consolidating biomedical research output. They mentioned that particular areas of research could benefit, such as tropical health, indigenous health and other regional priorities. Heightening opportunities for research collaboration is another benefit raised by the librarians. (Focus group, health sciences librarians, 28 participants, Adelaide).

A key strength of an Australasia PMC for biomedical researchers specifically relates to opening up more full-text manuscripts linked to research data. A Head biomedical researcher for example, indicated that having one site would make the data to be richly annotated and discoverable to allow researchers to download large sets and mine the content. (Interview participant, biomedical researcher, male, Victoria).

A librarian commented that there are groups that do not have access to subscription journals, proprietary bibliographic databases and other collections, such as general practitioners, clinicians outside the state and territory health service, private industry and not-for-profit community groups who would benefit significantly (Interview participant, senior librarian, female, New South Wales). According to the focus group participants, an Australasian PMC would provide ease of access and remove obstacles to full text papers.

All of the biomedical researchers interviewed indicated the importance of using primary research outputs. The clinical academics interviewed did use primary literature though commented that many of their peers did not. A participant commented that lack of access to research resources is a huge problem for some clinicians and pointed out that some do not even know how or where to find the research output (Interview participant, senior executive & biomedical researcher, female, Victoria). A hospital librarian highlighted the importance of searching the primary studies and non-commercial publications for research on redesign of service delivery and health technology applications. She commented that, “*health technology applications is another area where we go back to the primary literature usually indexed by MEDLINE*.” (Interview participant, senior librarian, female, New South Wales).

According to the librarians “*PubMed is at the base of the Evidence Based Healthcare pyramid and without that you cannot build upon the rest of the pyramid to achieve higher quality clinical information resources*.” (Interview group, health sciences librarians, 28 participants, South Australia).

#### Weaknesses

Findings about the opinions on usage of full text research articles was uneven. Clinicians and biomedical researchers interviewed mentioned that the sheer volume of primary research outputs and the work required to synthesize papers is a major reason why clinicians do not read primary papers. A senior executive and biomedical researcher claimed that they discourage clinicians from reading the raw evidence. Based on the vast amount of subscription and other content available from libraries and societies, a PMC maybe of limited value to many Australasian healthcare practitioners. An allied health practitioner claimed, “*MEDLINE is exclusive and doesn’t cover enough of the allied health sciences*.” (Hospital clinician/allied health practitioner, female, Victoria).

A clinician responded that they would not use a PMC, though indicated they did use Google Scholar for answering clinical queries. The first Google Scholar landing page will usually retrieve papers from PMC, so inadvertently many clinicians already rely upon PubMed and PMC.

A repository manager explained that in Australia principles and practices of repository interoperability need to occur in order to avoid the duplication of effort taking place throughout institutional repositories. Based on the investment in repositories, according to an institutional repository manager, Australian researchers appear to be ambivalent about open access publishing in gold and green modes; this is demonstrated by researchers’ lack of enthusiasm to submit ‘green’ approved versions of their research manuscripts to institutional repositories in response to funder and institutional open access policies [16].

Opportunistic predatory journals give open access publishing a bad reputation [17]. Most of the interviewees raised the importance of safeguarding the high standards traditional publishers have achieved over hundreds of years.

Two of the senior executives interviewed argued that Australia simply lacks the funding for PMC type projects; one executive stated, “*We don’t have Wellcome Trust funding in Australia*.” (Senior executive, female, Canberra). Biomedical researchers and a clinician raised the point that half of the content in journals is not reproducible and that this is a strong weakness of the published biomedical literature; this weakness only heightens the need to manage knowledge more effectively.

#### Opportunities

Health sciences librarians supported the notion that an Australasia PMC could mirror and contribute to PMCI and include biomedical content from Australian and New Zealand national libraries. The participants confirmed that the content in an Australasia PMC could include preprints, guidelines, government reports, patents, books, images and research data, such as the world clinical trial registries. An allied health practitioner argued that a PMC presents an opportunity to remove biomedical research out of silos, link to quality sites, and other core resources. The greater accessibility of resources would aid research engagement and provide an avenue for obtaining evidence of impact, which are priorities for research evaluation, such as the Excellence in Research for Australia process. A further possibility is the establishment of a regional network of medical libraries, to collaborate with a PMC; the US National Library of Medicine has achieved this, with membership of over 6,500 libraries in their support network [18].

There is potential to leverage a PMC for hospitals and health care organisations that do not have research repositories or the expertise to set them up. According to a senior hospital library manager: “*If there was an Australasia PMC harvesting citations it may even take away the need for every small health service to have their own and this would be excellent*.*”* (Interview participant, senior librarian, female, New South Wales). For researchers not affiliated with a university, an Australasia PMC would provide a suitable outlet to make their research openly available.

PMC can help make research more discoverable, for example, PMC contains the largest proportion of articles with open access copies, based on a sample of articles published since 2009 [19]. Systems such as PMC provide infrastructure to discourage authors from signing away their copyright to the publishers, who resell it to agencies that have funded it in the first place. User education on Creative Commons and promotion of the system to upload manuscripts become a possibility.

Bringing together suitable authorities to determine system protocol, screen quality resources and processes based on international principles, is a benefit of managing a regional PMC. A Head biomedical researcher proposed that an Australasia PMC could be a trusted site for promulgating significant research developments that are notable for healthcare practice.

Researchers interviewed described the opportunity for an Australasia PMC to be a single portal through which to open federated datasets and a means to get beyond restrictive journal subscription paywalls. A leading biomedical researcher explained that richly annotated content would ensure discoverability and provide researchers with datasets for mining content. This effort would complement the work currently underway to develop living systematic reviews and guideline creation [20].

Based on the EBHC pyramid design, researchers work down the levels of this model to seek relevant output to meet their research needs. Some biomedical researchers expressed difficulties with mastering specialised language for searching repositories, such as Boolean logic. Establishment of an Australasia PMC presents an opportunity to develop a repository system with greater search and retrieval precision. It may also be possible to tackle the indexing of studies whereby the research output is unreliable or not reproducible.

The National Library of Australia (NLA) has responsibility for making national digital content available, including Australian health and medical journals, books and reports that are of potential relevance to an Australasia PMC [21]. Using the NLA’s sophisticated data aggregation processes records from university repositories could be transferable to a PMC from the NLA’s Trove system.

Establishment of an Australasia PMC may be a means to transfer some of the traffic away from the ‘Wild West’ sites, such as *Sci-Hub*, where the pirating of research papers takes place and *ResearchGate* where users at times ignore or misunderstand copyright restrictions (Interview participant, senior repository manager, male, Victoria).

#### Threats

To become a node of PMCI requires a permanent commitment to maintain a digital archive. The commitment to open up the results of research that are publicly funded has been extensively debated and resulted in open access policies by the ARC and the NHMRC [22,23]. An interview with a senior executive leader in open access provided positive endorsement for an Australasia PMC on the basis that a PMC is a proven framework and as he described “only mundane matters such as how it would be financially supported and the longevity commitment would need to be sorted out.” (Interview participant, senior executive, leader in open access, male, Queensland).

A Head biomedical researcher, described clinical practice as “*incredibly complex and more complex with the passing of time as it tries to deal with multi-diseases in the same patient*, *who are treated with multiple different systems for looking after the different diseases*. *What might be right for a patient in a clinical trial in South Africa may be very wrong for another patient with a different set of problems*, *who happens to live in outback Australia*. *An intelligence system to screen and look at the data*, *so you can ask meaningful questions of it*, *so you are not limited to just searching a topic is required*.” (Interview participant, Head, biomedical researcher, male, Queensland). An Australasia PMC is not an immediate panacea to the information overload challenges, although it is a positive way to commence working cooperatively to contribute to a world medical library for long-term needs.

There are significant challenges in pioneering developments such as an Australasia PMC. It requires leaders from a range of fields to work together, to develop policies and infrastructure. There is the threat of organisations’ inability to work together to establish and maintain a PMC.

Based on the large number of clinicians with academic titles, entitling them with access to university library collections, the present lack of motivation from this sector in changing the scholarly publishing system is another threat.

## Discussion

Most interviewees agreed with exploring the opportunity to become a partner with Europe PMC as a means to capitalise on the strengths of PMCI. This is an attractive option given efficiencies gained from Europe PMC are reported to be worth around £1 billion per annum worldwide, or 20 times the direct operational cost [24].

Further research on the lessons learnt from PMC Canada and Europe PMC can inform an investigation on the suitability for an Australasia PMC. Collaboration with the National Libraries of Australia and New Zealand to achieve an Australasia PMC may be possible.

There are no formal measures of university compliance with open access requirements of funding bodies in Australia. PMCI has been able to achieve high levels of compliance, as publishers submit author content to the repository in most cases. This removes the submission burden from the researcher and may be a key reason the PMC model is effective. It will be important for Australasia funding bodies, such as the NHMRC, ARC and the Health Research Council of New Zealand to consider the opportunities made possible from investing in a permanent and sustainable PMC.

The closure of PMC Canada suggests that some of the challenges for sectors within the healthcare industry, government and universities, to work together are difficult to resolve, though the success of establishing a PMC in the European region is counter to the experience in Canada [3,25].

An agreement with Europe PMC to establish an Australasia PMC may be the viable solution to overcome the identified threats of lack of commitment from a national body and inability to strike a deal with the US NLM.

**Fig 1:**
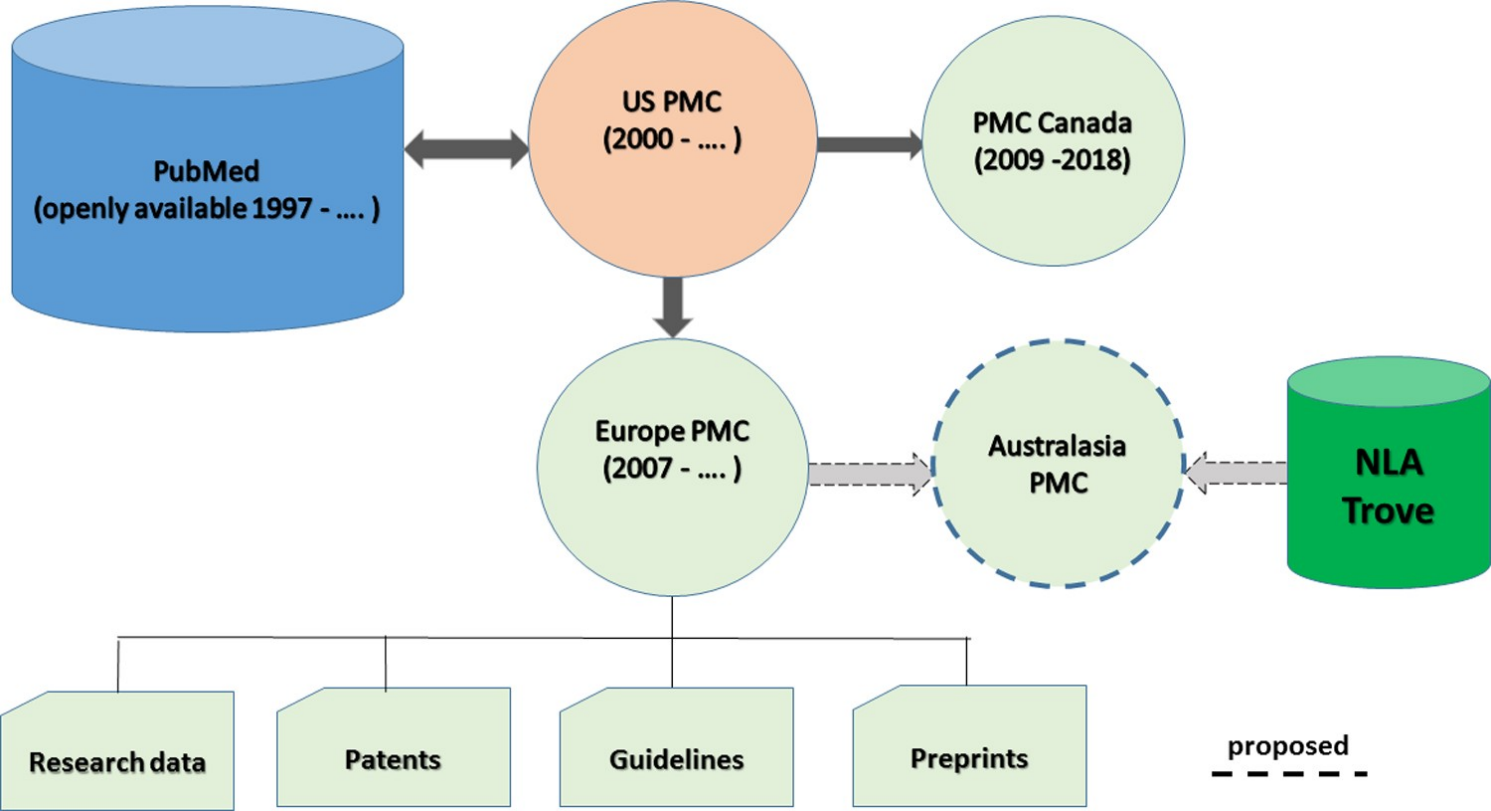
Diagram of PMC international with a potential Australasia PMC.

Improving Australasian biomedical knowledge management processes may be possible from adopting a PMC for consolidated storage, retrieval and transfer processes of research linked to its underlying data. This in turn could put regional biomedical research under a stronger spotlight and potentially lead to improvements with research quality. The amount of content available from an open consolidated PMCI repository, in particular for data and text mining, will grow if Europe PMC and an Australasia PMC can combine forces (Fig 1 Diagram of PMC International with a potential Australasia PMC). This in turn contributes to the range of bio-reports that are possible, with flow-on benefit to industry and those groups often excluded from public research due to journal subscription paywalls.

An Australasia PMC fits with the objective of making Australia’s publicly funded research outputs F.A.I.R. (Findable, Accessible, Interoperable, Reusable) [26]. It presents opportunities to enhance the clinical research cycle process and optimise Australasian biomedical research through the establishment of a permanent archive available to all.

### Study limitations and future research

PMCI comprised the US PMC, Europe PMC and PMC Canada, as previously mentioned in this paper, when the interviews and the focus group took place. Views on the potential of an Australasian PMC obtained from participants were not as an alternative to the existing solution that is institutional repositories, but as a chance to explore the Strengths Weaknesses, Opportunities and Threats of a PMC in the region. As this paper reports on Cycle One of an action research project, investigating more effective biomedical knowledge management, it is possible that solutions other than an Australasia PMC will develop in the later cycles.

Another limitation of this research is the focus on biomedicine. The major reasons for the focus on biomedicine is firstly because the field generates a prolific amount of world research output and Australia is in the top twenty countries with the most biomedical publications [27,28]. Additionally, in matters of life and death, access to health research output should have no barriers. This research builds on the worldwide effort to reduce barriers, in particular paywalls to health knowledge. There are internationally established classification and information management schemes available to build upon. Further to this, an open biomedical repository proof of concept can potentially expand to other disciplinary areas to achieve a future regional multi-disciplinary repository.

Cycle Two of our research is the conceptual design of an Australasia PMC based on knowledge management theories and informed by interviews with PMCI leaders. Assessing the design for an open biomedical repository, such as an Australasia PMC, will complete this research project.

## Conclusion

The key opportunities for a potential Australasia PMC identified by this research are greater discoverability and accessibility of biomedical regional research output, greater sharing of repository expertise, consolidation, improved copyright compliance, data-set integration and an increased provision of mineable and reusable content. The opportunity for an Australasia PMC to overcome threats, such as the present adequacy of existing repository and information resource access and to address the problem of limited available funding to ensure longevity of PMC Australasia remains to be tested.

The adoption of formalised knowledge management processes could potentially result in significantly improved biomedical information systems [29]. There is a great opportunity for a body such as the NHMRC to take a leadership role in consolidating the present fragmented approach to the management of biomedical information by linking the research output to evidence of impact and improvements for society.

A major reason why PMC Canada went offline in 2018 was due to the growth in Canadian institutional repositories and other technology systems that supersede that of PMC. Even so, institutional repositories in Australia on average achieve low levels of compliance with funder open access policies, whereas due to effective processes the US National Institutes of Health and Wellcome Trust achieve compliance rates around 90% using the US PMC and Europe PMC repositories respectively [16,30].

As a senior biomedical researcher concisely sums up, “*As we increase Open Access to make knowledge more accessible and if an Australasia PMC does this it would be worthwhile*. *It is not just about clinicians accessing an Australasia PMC*. *The ways it would contribute are diverse*, *an Australasia PMC would be accessible to consumers and this is important*.” (Interview participant, Head biomedical researcher, male, Queensland). A blueprint for a sustainable Australasia PMC is the way forward and a goal for the Australasia PMC Working Group to pursue.

## Supporting information

S1 File Interview and focus group questions. (PDF)

S2 File Explanatory statement for researchers. (PDF)

S3 File Explanatory statement for library staff. (PDF)

S4 File Australia PMC briefing document. (PDF)

S5 File De-identified spreadsheet of participant interview transcripts. (XLSX)
