# Supplementary material for: With open science gaining traction, do we need an Australasia PubMed Central (PMC)? A qualitative investigation
Source: PLoS One. 2019 Feb 22;14(2):e0212843. doi: 10.1371/journal.pone.0212843 (PMC6386259; doi:10.1371/journal.pone.0212843)
Supplement: S1 File — (PDF) [file pone.0212843.s001.pdf]

Appendix 1:

INTERVIEW QUESTIONS:

The below questions are used to frame the discussion. Being a MEDLINE/PubMed searcher was not a requirement for the interview.

1. Please describe the nature and extent of your experience with MEDLINE, PubMed and PubMed Central (PMC)

2. What platform do you use to access MEDLINE?

a. Google scholar
b. OVID
c. EBSCO
d. SciFinder
e. Embase.com
f. PubMed (National Library of Medicine)
g. Europe PMC
h. PMC Canada
i. Other

3. How frequently do you access MEDLINE/PubMed?

a. Daily
b. Weekly
c. Monthly
d. A few times a year
e. Other

4. How important are resources such as MEDLINE/PubMed/PubMed Central to filling gaps in clinical knowledge?

5. Would an open access repository, such as Australia PMC, contribute to clinical practice?

6. Based on your experience and or reading of the Australia PMC Briefing Document please explain what you perceive to be any potential value of an Australia PMC to the medical and health sciences research community?

7. Do you have any other feedback? Such as risks and drawbacks of an Australia PMC
