# Supplementary material for: With open science gaining traction, do we need an Australasia PubMed Central (PMC)? A qualitative investigation
Source: PLoS One. 2019 Feb 22;14(2):e0212843. doi: 10.1371/journal.pone.0212843 (PMC6386259; doi:10.1371/journal.pone.0212843)
Supplement: S2 File — (PDF) [file pone.0212843.s002.pdf]

MONASH University

EXPLANATORY STATEMENT

Researchers and Clinicians

Project: Feasibility of establishing an Australia PubMed Central: Phase One

Chief Investigator
Professor Frada Burstein
Leader, Health ICT theme
Faculty of IT
Monash University
P: +61 3 9903 2011
E: frada.burstein@monash.edu

Researcher
Dr Kerry Tanner
Adjunct Senior Research Fellow
Faculty of IT
Monash University
P: 61 3 9903 2551
E: kerry.tanner@monash.edu

PhD Researcher
Ms Lisa Kruesi
Faculty of IT
Monash University
M: 0412 655 787
E: lisa.kruesi1@monash.edu

As a leader in your field you are invited to take part in this study. Please read the **Explanatory Statement and Australia PubMed Central (PMC) Briefing Statement** before deciding whether or not to participate in this research. If you would like further information regarding any aspect of this project, you are encouraged to contact the researchers via the phone numbers or email addresses listed above.

We are seeking stakeholder endorsement to investigate and design an Australia PubMed Central (PMC) prototype. Like its counterpart, Europe PMC, an Australia PMC would potentially include abstracts, articles and links to research data from anywhere in the world. It could also include open access articles from Australian peer reviewed biomedicine and health journals not indexed by MEDLINE, making it unique. The full scope of content from the US PubMed and PMC would be available from an Australia PMC and delivered through a single search platform.

What does the research involve?

A briefing statement about Australia PMC is included as an attachment, along with this statement.

If you agree to participate, Lisa Kruesi will phone your office to make an appointment to interview you. The interview can be held at a time convenient to you and will involve discussion of eight questions. It should only take 45 minutes of your time.

If you are willing to participate please sign and return the Consent Form also included as an attachment.

Why were you chosen for this research?

As a leader with significant experience you have been chosen for this research. Your contact details were obtained from your organisation's website.

Voluntary nature of participation

Participants retain the right to withdraw from this investigation at any stage of the research. If the participant has chosen to provide an anonymous questionnaire response it will not be possible to withdraw the data once they have submitted a response. If the participant is identifiable and they choose to withdraw from the study, their response can be withdrawn within one month of their submission.

Possible benefits and risks to participants?

Participation in this project will not have any immediate benefits to you. However, you will be making a major contribution to answering an important research question on the need for an Australia PMC. If an Australia PMC were to proceed in the future there would be many benefits, such as:

- Opportunity to avoid duplication of effort and time savings based on consolidation of manuscripts in one biomedical repository
- Access to an international repository system at institutes, hospitals and healthcare services that do not have such systems or expertise to develop such systems. For example, a senior library manager working at a major teaching hospital in Western Australia commented that: "I know the universities are pretty well served by their own repositories but it is a big issue for purely hospital or research institute based research – we don't have repositories and I know I am asked about it here. Seems mad to have lots and lots of little repositories set up (sustainability is a big issue) when it would be so much more efficient if we all plugged into a single national one."
- Importance of open access to research and a need for a repository for peer-reviewed journal papers funded partly or in whole by the ARC/NHMRC/NIH

- Avenue for a comprehensive repository of Australian medical and health sciences research for discovery and merging of publication records with other world PMC sites
- Opportunity to use the site as an outlet to record the translation and adoption of evidence into practice
- Means to preserve Australian health research and link to associated data
- The site would be a means to help determine Australian medical research areas, identify experts and champions for collaboration and a site to obtain studies by Australian researchers
- Potential future resource for answering clinical questions and accessing journal articles
- Avenue to identify potential collaborators and experts

Possible or reasonably foreseeable risks of harm to the potential participants:

- There are no foreseeable risks of harm to potential participants from participation in the research
- The topic is not controversial
- Participants' details will be kept confidential

Confidentiality

This matter is not of a highly sensitive nature. Participants can request that their responses be kept anonymous and confidential. Based on this request their correspondence will be suitably labelled and saved on a password protected network. Responses labelled confidential will not be transcribed within the final thesis or associated papers.

Storage of data

The data we collect will only be accessed by the named researchers. All data will be securely stored at the researchers' institutions and will be stored centrally at Monash University after the completion of the research. Audio recordings of interviews will be destroyed after the transcripts have been approved. With your approval, data will be deposited in the Monash University Research Repository. Otherwise the data will be securely destroyed five years after the completion of the project to comply with Monash University guidelines.

Use of data for other purposes

With your permission, we would like to use the data we collect in future research. Only aggregate de-identified data will be made available for other research and only for research where ethics approval has been granted.

Results

It is anticipated that the PhD thesis will be made openly available on the Monash University thesis website.

Complaints

Should you have any concerns or complaints about the conduct of the project, you are welcome to contact the Executive Officer, Monash University Human Research Ethics (MUHREC):

Executive Officer
Monash University Human Research Ethics Committee (MUHREC)
Room 111, Chancellery Building E,
24 Sports Walk, Clayton Campus
Research Office
Monash University VIC 3800

Tel: +61 3 9905 2052   Email: muhrec@monash.edu

Thank you,

Prof Frada Burstein

Summary of Invited Actions:

1. **Please read the Explanatory Statement and Australia PubMed Central (PMC) Briefing Statement**

2. **Please sign and return the Consent Form**
