# Supplementary material for: With open science gaining traction, do we need an Australasia PubMed Central (PMC)? A qualitative investigation
Source: PLoS One. 2019 Feb 22;14(2):e0212843. doi: 10.1371/journal.pone.0212843 (PMC6386259; doi:10.1371/journal.pone.0212843)
Supplement: S4 File — (PDF) [file pone.0212843.s004.pdf]

Australia PubMed Central (PMC): Briefing Statement

What is the relationship between MEDLINE, U.S. PubMed and PMC?

MEDLINE is the U.S. National Library of Medicine® (NLM) major bibliographic database with over 25 million references to journal articles on biomedicine and health. PubMed is a free citation index, produced by the National Center for Biotechnology Information (NCBI) at the NLM, which contains MEDLINE plus NLM's database of citations and abstracts. PubMed Central® (PMC) is a free full-text archive of biomedical journal literature. PMC provides content deposited by participating publishers and author manuscripts submitted to comply with the NIH public access policy. Some PMC journals are also MEDLINE journals, and there are links between corresponding citations in PubMed and the full text in PMC. See Figure 1 below.

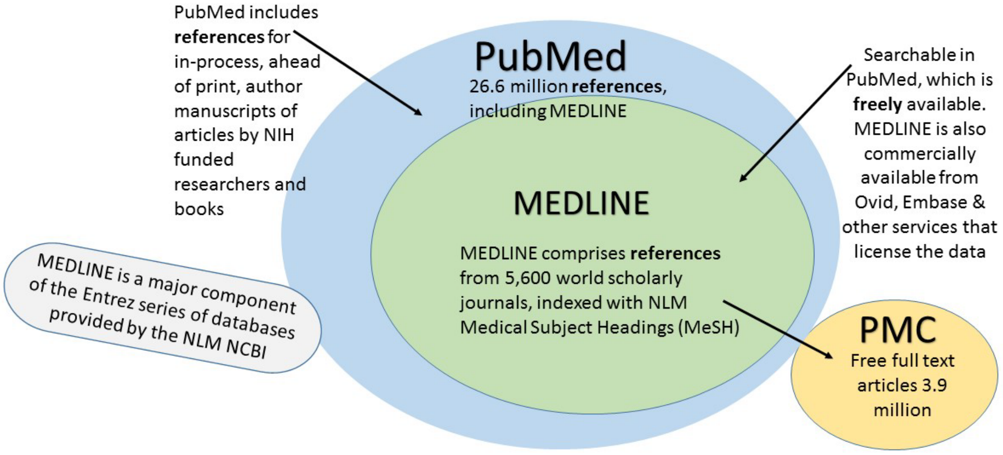

Figure 1 Source: https://www.nlm.nih.gov/pubs/factsheets/medline.html (Graphic by L. Kruesi, 2016)

What is an Australia PMC?

The Australia PMC's main purpose would be to establish a consolidated repository of openly available biomedical and health sciences articles by Australian researchers. An Australia PMC, like its counterpart, Europe PMC, can potentially include abstracts, articles and links to research data from anywhere in the world, and would not be limited by funder or geographical location. It could also include open access articles from Australian peer reviewed biomedicine and health journals not indexed by MEDLINE, making it unique. The full scope of content in the USA PubMed, MEDLINE and PMC would be available from an Australia PMC and delivered through a single search platform. An example of the content in the Europe PMC follows in Figure 2.

Why an Australia PMC?

It is an opportunity to:

- Create a comprehensive Open Access repository of papers by Australian health and medical researchers
- Address the fragmented and incomplete access to Australian health research publication records

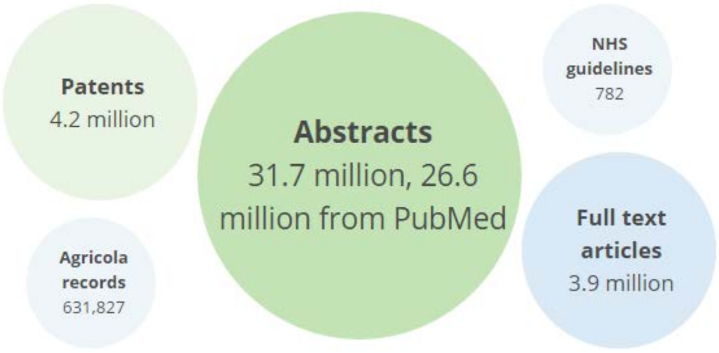

Figure 2 Diagram of the Europe PMC taken from https://europepmc.org/About

Why an Australia PMC? …..

- Overcome the significant amount of duplicated effort underway in Australian universities to collect publications for repositories in order to meet funding bodies' open access mandates
- Provide a repository system for sites without or with limited access, such as Australian medical institutes, hospitals and healthcare sites
- Leverage existing infrastructure to develop a comprehensive repository for peer-reviewed journal papers funded by the ARC/NHMRC
- Merge Australian articles with other world PMC sites
- Preserve Australian health research and associated data for present and future generations
- Become a node of the USA NLM PubMed and contribute internationally to the creation of a nationally owned, quality database of medical and health sciences content

How?

A steering committee, comprising members from Monash University, The Association of Australian Medical Research Institutes, University of Melbourne and the University of Queensland, will prepare a proposal recommending a suitable model and design of an Australia PMC prototype. The proposal will take into consideration contractual requirements of the NIH/NLM/NCBI and include funding recommendations. A prototype design and recommendations would be made available for consideration in late 2017.

Need?

This research will explore the importance of an Australia PMC repository from a knowledge management perspective. The research will be based on the Haynes' 6S pyramid model of the six hierarchical levels of access to the best clinical evidence (1). Knowledge management principles, which include tacit as well as explicit knowledge, will be integrated within the Haynes' model to enhance the understanding of the uptake of clinical evidence and transfer into practice.

November 2016

(1) DiCenso, A., Bayley, L., & Haynes, R. B. (2009). Accessing pre-appraised evidence: fine-tuning the 5S model into a 6S model. *Evidence based nursing, 12*(4), 99-101.

Recently updated:

Alper, B. S., & Haynes, R. B. (2016). EBHC pyramid 5.0 for accessing preappraised evidence and guidance. *Evidence Based Medicine, 21*(4), 123. doi: 10.1136/ebmed-2016-11044
